# Intramembrane proteolysis of an extracellular serine protease, epithin/PRSS14, enables its intracellular nuclear function

**DOI:** 10.1186/s12915-020-00787-3

**Published:** 2020-06-03

**Authors:** Youngkyung Cho, Sang Bum Kim, Jiyoon Kim, An Vuong Quynh Pham, Min Ji Yoon, Jeong Hwan Park, Ki-Tae Hwang, Dongeun Park, Yongcheol Cho, Moon Gyo Kim, Chungho Kim

**Affiliations:** 1 Department of Life Sciences, Korea University, Seoul, 02841 Republic of Korea; 2 School of Biological Sciences, Seoul National University, Seoul, 08826 Republic of Korea; 3 Present address: Severance Biomedical Science Institute, Yonsei University College of Medicine, Seoul, 03722 Republic of Korea; 4 Department of Pathology, Seoul Metropolitan Government Seoul National University Boramae Medical Center, Seoul, 07061 Republic of Korea; 5 Department of Surgery, Seoul Metropolitan Government Seoul National University Boramae Medical Center, Seoul, 07061 Republic of Korea; 6 Department of Biological Sciences, Inha University, Incheon, 22212 Republic of Korea

**Keywords:** Epithin/PRSS14, Regulated intramembrane proteolysis, Transcriptional regulation, Metastasis

## Abstract

**Background:**

Epithin/PRSS14, a type II transmembrane serine protease, is an emerging target of cancer therapy because of its critical roles in tumor progression and metastasis. In many circumstances, the protease, through its ectodomain shedding, exists as a soluble form and performs its proteolytic functions in extracellular environments increasing cellular invasiveness. The seemingly functional integrity of the soluble form raises the question of why the protease is initially made as a membrane-associated protein.

**Results:**

In this report, we show that the epithin/PRSS14 intracellular domain (EICD) can be released from the membrane by the action of signal peptide peptidase-like 2b (SPPL2b) after ectodomain shedding. The EICD preferentially localizes in the nucleus and can enhance migration, invasion, and metastasis of epithelial cancer when heterologously expressed. Unbiased RNA-seq analysis and subsequent antibody arrays showed that EICD could control the gene expression of chemokines involved in cell motility, by increasing their promoter activities. Finally, bioinformatics analysis provided evidence for the clinical significance of the intramembrane proteolysis of epithin/PRSS14 by revealing that the poor survival of estrogen receptor (ER)-negative breast cancer patients with high epithin/PRSS14 expression is further worsened by high levels of SPPL2b.

**Conclusions:**

These results show that ectodomain shedding of epithin/PRSS14 can initiate a unique and synchronized bidirectional signal for cancer metastasis: extracellularly broadening proteolytic modification of the surrounding environment and intracellularly reprogramming the transcriptome for metastatic conversion. Clinically, this study also suggests that the intracellular function of epithin/PRSS14 should be considered for targeting this protease for anti-cancer treatment.

## Background

Protease serine 14 (PRSS14), a gene of the 14th serine protease, was initially named as epithin [1] and is also known as Matriptase [2], MT-SP1 [3], or ST14 [4]. It is a prototypic member of type II transmembrane serine protease containing its relatively short cytoplasmic tail in the N-terminal and its extracellular protease part in the C-terminal regions [5, 6]. Many studies have shown that epithin/PRSS14 is upregulated in a variety of human epithelial cancers [7] and is tightly linked to higher tumor grades and poorer survival of patients [8–11]. Recently, we reported from the analysis of public databases of patients with breast cancer that epithin/PRSS14 is a prominent prognosis marker for patients with estrogen receptor (ER)-negative breast cancer [12]. The role of epithin/PRSS14 in metastasis and survival has been evaluated in multiple animal studies. Impaired metastasis was observed when cancer cells with low epithin/PRSS14 expression were injected [13]. In contrast, the high expression of epithin/PRSS14 enhanced angiogenesis, invasive tumor growth, and metastasis [14, 15]. Transgenic mice expressing epithin/PRSS14 driven by the K5 promoter developed spontaneous squamous carcinoma and metastasis [16]. When MMTV-PyMT mouse models were crossed with a matriptase hypomorphic model, expressing very little epithin/PRSS14 expression, the animals lived longer with less tumor burden [17]. These studies clearly show the important role of epithin/PRSS14 in tumor progression, thus making it a promising target for anti-cancer therapy [6].

Our previous study showed that epithin/PRSS14 is synthesized as a single-chain zymogen and is cleaved between Gly149 and Ser150 in the SEA (sperm protein, enterokinase, and agrin) domain to produce two fragments, the N-terminal fragment (NTF) and the C-terminal ectodomain containing the protease domain (named as Epi-S), which are non-covalently associated on the membrane [18]. The cleavage in the SEA domain is suggested to be mediated by self-cleavage mechanism through attack of the hydroxyl group of the serine residue on the neighboring glycine carbonyl group, without involvement of action of other proteases [19]. Further cleavage at Arg186, Lys189, or Lys204 releases most of the ectodomain, leaving the NTF and the small ectodomain fragment on the plasma membrane. This shedding is initiated by various stimuli, such as the serum [20], phorbol 12-myristate 13-acetate (PMA) [21], transforming growth factor-β (TGF-β) [22], hypoxia [23], and acidic conditions [24], which possibly activates its sheddases, TACE/ADAM17 [22, 25] and/or serine protease [24]. Consistently, we have shown that knocking down TACE inhibits the ectodomain shedding of epithin/PRSS14 [22, 25]. The released extracellular domain may extend the proteolytic effects of epithin/PRSS14 to a larger degree modulating the extracellular microenvironment in favor of cellular invasion. If this is the case, why the protease is made as an integral membrane protein and has to be released by an additional step is of question. In this regard, the possible role of NTF of epithin/PRSS14 was investigated.

Here, the intracellular function of NTF is presented. Specifically, it was found that NTF of epithin/PRSS14 left on the plasma membrane upon ectodomain shedding can be further processed and liberated from the membrane by intramembrane proteolysis. It was also found that the epithin/PRSS14 intracellular domain (EICD) can promote migration, invasion, and cancer metastasis, possibly through transcriptional changes. The intracellular function of the protease would be locked until ectodomain shedding occurs, and when unlocked, it can potentiate cellular behaviors mediated by the released extracellular protease. Therefore, we suggest that its existence as an integral membrane protein may be required for the cooperative functions of extracellular and intracellular domains in a synchronized manner at the time of ectodomain shedding.

## Results

### The intracellular domain of epithin/PRSS14 is detected in the nucleus

**Fig. 1:**
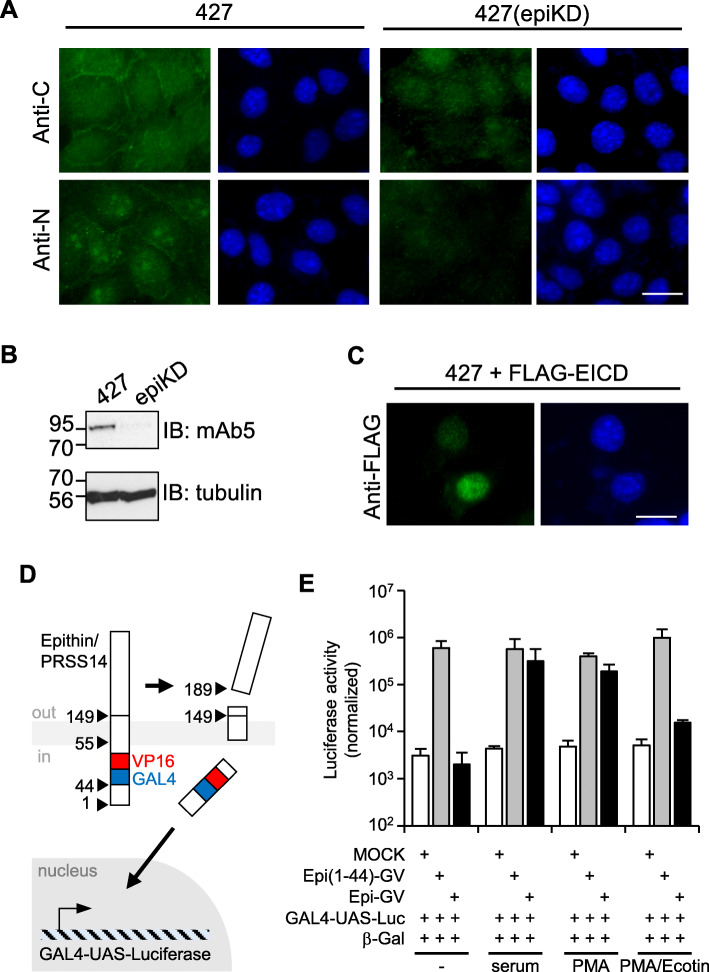
Nuclear localization of epithin/PRSS14 intracellular domain. **a** Epithin/PRSS14 in 427.1.86 cells (427) or epithin/PRSS14-knockdown 427(epiKD) cells were stained green using antibodies against its extracellular C-terminal parts (anti-C) and intracellular parts (anti-N). Nucleus staining with DAPI (blue) is also shown. Scale bars, 20 μm. **b** The Western blot analyses of epithin/PRSS14 in 427 cells and 427 (epiKD) cells are shown. **c** The 427 cells transfected with FLAG-EICD were stained using anti-FLAG antibody (green) and DAPI (blue). Scale bars, 10 μm. **d** Schematic diagram of the assay to validate cleavage of epithin/PRSS14. **e** The 427 cells were transfected as indicated and deprived of serum before stimulation with 10% FBS, 10 μM PMA, or 10 μM PMA plus 7 mg/ml ecotin for 1 h. Luciferase activity under each condition was normalized against β-galactosidase (β-gal) activity and is shown as a bar graph. Error bars indicate the standard errors of the means (*n* = 3)

When 427.1.86 thymoma cells (427 cells) expressing epithin/PRSS14 endogenously were stained with an antibody generated against the ectodomain of epithin/PRSS14 (anti-C), a membrane staining pattern was observed preferentially in the cell-cell contacts (Fig. 1a). However, when stained with an antibody against the intracellular domain (anti-N), a punctate staining pattern in the nucleus was also observed (Fig. 1a). These staining patterns were not observed in the 427(epiKD) cells (Fig. 1a), a cloned cell line derived from epithin/PRSS14 targeting shRNA-transfected 427 cells (Fig. 1b), showing specificities of the staining. In addition, the intracellular domain tagged with the FLAG epitope was also localized in the nucleus (Fig. 1c). These findings raise the possibility that the epithin/PRSS14 N-terminal region may detach from the plasma membrane and translocate to the nucleus.

To test this, the epithin/PRSS14-GAL4-VP16 construct (Epi-GV) was generated in which the GAL4-VP16 is inserted into the cytoplasmic tail of the protease (Fig. 1d) and whether it can induce transcription of the luciferase gene under a GAL4 upstream activating sequence (GAL4-UAS-luciferase) was examined. Surface expression of the GAL4-VP16-inserted epithin/PRSS14 construct was confirmed by flow cytometry (Additional file 1: Figure S1A and S1B). Since transcription of the luciferase gene is only possible when the GAL4-VP16 can freely move to the nucleus, this system allows sensitive detection of a possible liberation of the cytoplasmic tail of membrane proteins [26]. As a control, the intracellular domain part of epithin/PRSS14 was fused to GAL4-VP16, Epi (1-44)-GV, which can mimic a liberated intracellular domain of the fusion protein. When co-transfected with GAL4-UAS-luciferase reporter into 427 cells, EICD-GV largely induced luciferase activity in all conditions (Fig. 1e). In contrast, 427 cells transfected with Epi-GV showed increases in luciferase activity only when its ectodomain shedding was induced by treating serum or PMA (Fig. 1e). Moreover, the PMA-induced increase in luciferase activity was blocked by treating a known epithin/PRSS14 shedding inhibitor, ecotin [21]. Consistently, when serum-starved 427 cells were stimulated with PMA, the nuclear dots recognized by anti-N antibody were increased, while knocking down the epithin/PRSS14 expression by siRNAs suppressed the increase (Additional file 1: Figure S2A). Quantification analysis using an in-house Matlab code (Additional file 1: Figure S2B and S2C) confirmed significant increase of the nuclear dots in PMA-treated condition (Additional file 1: Figure S2D). Together, these results show that ectodomain shedding of epithin/PRSS14 can liberate EICD from the membrane.

### Ectodomain shedding of epithin/PRSS14 generates EICD

**Fig. 2:**
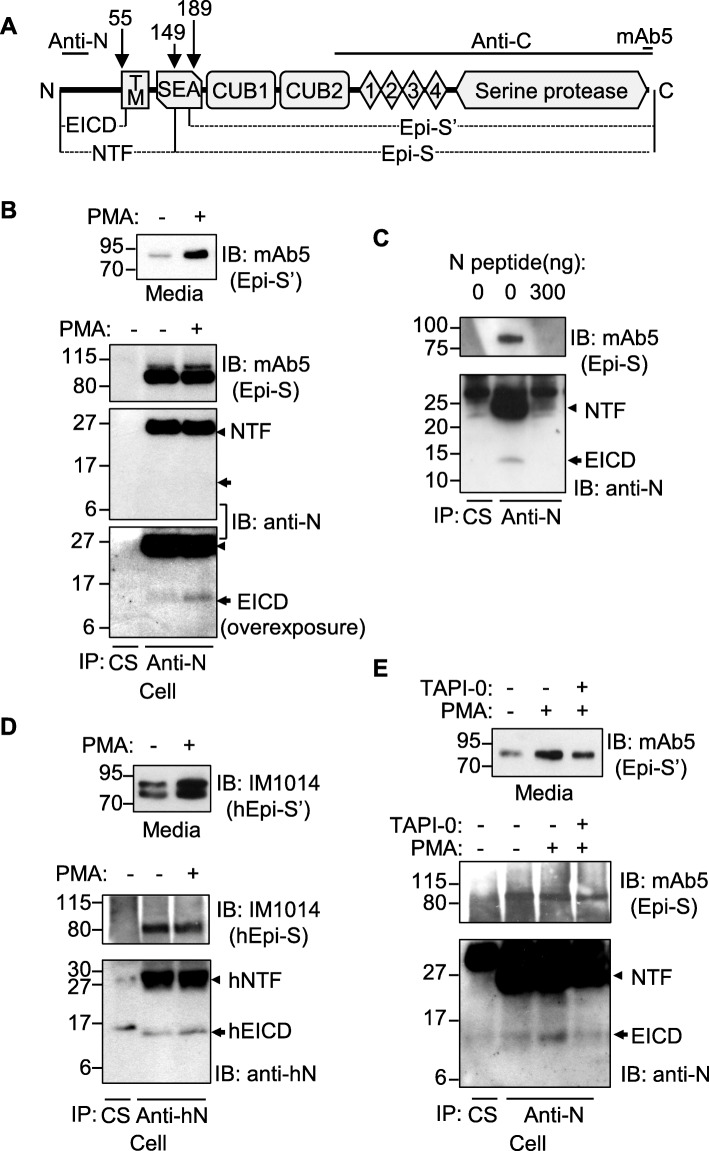
Ectodomain shedding induced the generation of the epithin/PRSS14 intracellular domain. **a** Schematic diagram of the epithin/PRSS14 domain structure, its possible proteolytic fragments, and epitopes of antibodies used in this study are illustrated. TM, transmembrane domain; SEA, sperm protein, enterokinase, and agrin domain; CUB1, CUB2, complement subcomponent C1r/C1s domain; 1, 2, 3, 4, low-density lipoprotein receptor class A repeats. **b** The 427 cells were treated with or without PMA and shed epithin/PRSS14 (Epi-S′) was analyzed using mAb5. Cell-bound epithin/PRSS14 (Epi-S), NTF, and EICD were detected by immunoprecipitation and subsequent Western blot with mAb5 and anti-N antibody under each condition. CS, control serum. **c** After the antigen of anti-N antibody (Gly12-Arg25) was added or left out of the immunoprecipitation reaction, Epi-S, NTF, and EICD in the anti-N antibody, immunoprecipitates were analyzed as in **b**. **d** The proteolytic fragment of human epithin/PRSS14 was analyzed using MCF7 human breast cancer cells. **e** The 427 cells were treated with PMA and/or TAPI-0, and each fragment was analyzed as in **b**. Arrowhead and arrow indicate NTF and EICD, respectively

To verify the existence of the EICD, the proteolytic fragments of epithin/PRSS14 in 427 cells were analyzed using various antibodies (Fig. 2a). As reported previously, about 5% of Epi-S in cells was shed and detected in media as Epi-S' upon PMA treatment (Fig. 2b, top panel). Immunoprecipitation and subsequent Western blot using the anti-N antibody detected the NTF (Fig. 2b). After more prolonged exposure of the same membrane, a smaller fragment was also detected with a molecular weight (~ 14 kDa) corresponding to the size of EICD in the immunoprecipitates from PMA-treated cells (Fig. 2b, bottom panel). The 14 kDa band disappeared by adding the antigen of anti-N antibody (Gly12-Arg25) to the immunoprecipitation reaction (Fig. 2c) and also by siRNA-mediated knocking down epithin/PRSS14 expression (Additional file 1: Figure S3A), confirming that the band was derived from epithin/PRSS14. Moreover, the 14 kDa band was preferentially observed in the nuclear fraction, but not in cytosolic fraction, of PMA-stimulated cells, while knocking down epithin/PRSS14 expression reduced the band intensity in the nuclear fraction again (Additional file 1: Figure S3B). In MCF7 human breast cancer cells known to express epithin/PRSS14, similar ~ 14 kDa proteolytic fragment was observed using the anti-N antibody raised against the human epithin/PRSS14 intracellular domain (Fig. 2d), indicating that the generation of the EICD may be the general phenomena in epithin/PRSS14-expressing cells. In agreement with the result from the GAL4-VP16 transcriptional system (Fig. 1e), the inhibition of shedding by the TACE inhibitor TAPI-0 reduced the EICD generation (Fig. 2e). Taken together, these data suggest that the ectodomain shedding of epithin/PRSS14 generates EICD.

### SPPL2b is responsible for the intramembrane proteolysis of epithin/PRSS14

**Fig. 3:**
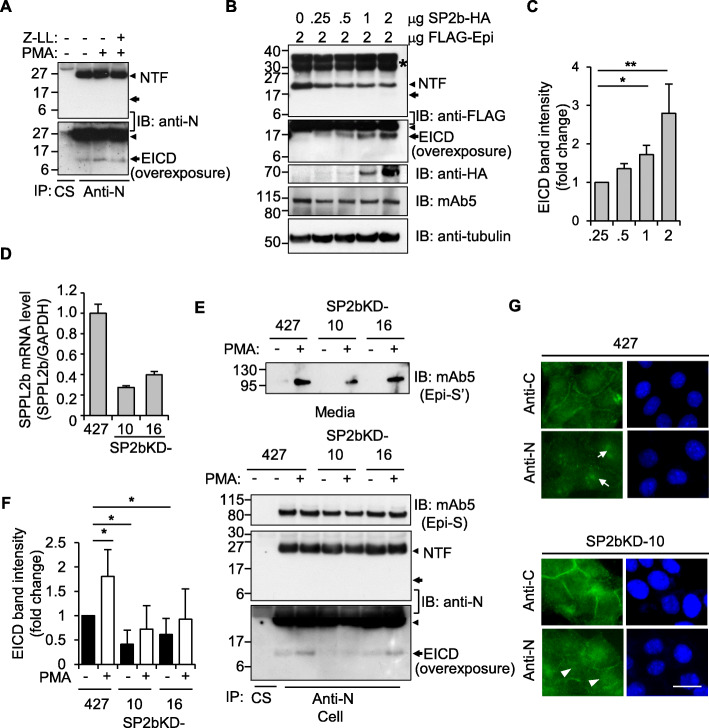
SPPL2b-mediated intramembrane proteolysis of epithin/PRSS14. **a** The 427 cells were treated with PMA and/or SPPLs inhibitor, 10 μM (Z-LL)_2_ketone and proteolytic fragments were analyzed as in Fig. 2b. **b** HEK293T cells transfected with FLAG-tagged epithin/PRSS14 and increasing amounts of SPPL2b-HA were analyzed by Western blot using the anti-FLAG antibody to detect NTF and EICD. The expression of SPPL2b and full-length epithin/PRSS14 were also confirmed by Western blot using anti-HA and anti-FLAG antibodies, respectively. Asterisk indicates uncharacterized fragments, presumably resulted from nonspecific cleavages, glycosylation, and/or other modifications of overexpressed epithin/PRSS14. **c** EICD band intensity was quantified and normalized against tubulin blot and is shown as a bar graph (*n* = 3). The error bar indicates SD. *p < 0.05, **p < 0.01 (unpaired two-tailed Student’s *t* test). **d** The mRNA levels of SPPL2b in SPPL2b-knockdown cell lines (SP2bKD-10 and -16) were detected by real time-PCR. The relative values were normalized to GAPDH signals, as shown in the graphs. Error bars indicate SEM. **e** Proteolytic fragments of epithin/PRSS14 were analyzed in the control and SP2bKD cells, as in Fig. 2b. **f** The average of normalized EICD band intensities from three independent immunoprecipitation experiments is shown as a bar graph (*n* = 3). The error bar indicates SD. *p < 0.05 (unpaired two-tailed Student’s *t* test). Arrowhead and arrow indicate NTF and EICD, respectively. **g** Localization of the N-terminal and C-terminal parts of epithin/PRSS14 were analyzed, as in Fig. 1a. Note that the nuclear localization of EICD (arrows) was reduced in SP2bKD-10 cells (arrowheads). Scale bars, 20 μm

These observations led to the hypothesis that EICD may be generated by the proteolytic cleavage occurring inside the membrane, known as regulated intramembrane proteolysis (RIP) [27]. Because the aspartyl protease SPPL family is responsible for the RIP of type II transmembrane proteins [28], the possible involvement of SPPLs in the production of EICD was investigated. First, treatment of an inhibitor of the SPPL family, (Z-LL)_2_ketone, reduced the PMA-induced increase in EICD formation (Fig. 3a). In contrast, DAPT, an inhibitor of γ-secretase responsible for the RIP of type I transmembrane protein, had no effect on the generation of EICD (Additional file 1: Figure S4). Second, expression of SPPL2b, the only member of an SPPL family known to be present in the plasma membrane [29], in HEK293T cells resulted in the generation of EICD from co-transfected epithin/PRSS14 in a dose-dependent manner (Fig. 3b and c). Third, in SPPL2b known-downed 427 cell lines, 427(SP2bKD)-10 and -16 (Fig. 3d), PMA-induced EICD generation decreased (Fig. 3e, bottom panel and 3F), while PMA-induced shedding occurred normally (Fig. 3e, top panel). Finally, the nuclear staining of epithin/PRSS14 was also diminished in 427(SP2bKD)-10 cells without alteration to membrane staining at the cell-cell contact (Fig. 3g and Additional file 1: Figure S5A). Consistently, the EICD band in the nuclear fraction observed in PMA-treated 427 cells was largely reduced in 427(SP2bKD) cells (Additional file 1: Figure S5B). Collectively, these results showed that SPPL2b may be an enzyme responsible for the cleavage of NTF left on the plasma membrane and the production of EICD. Nonetheless, we do not exclude a possible contribution of other member of SPPL family, e.g., SPPL2a, on the EICD production.

### EICD promotes cell migration, invasion, and metastasis

**Fig. 4:**
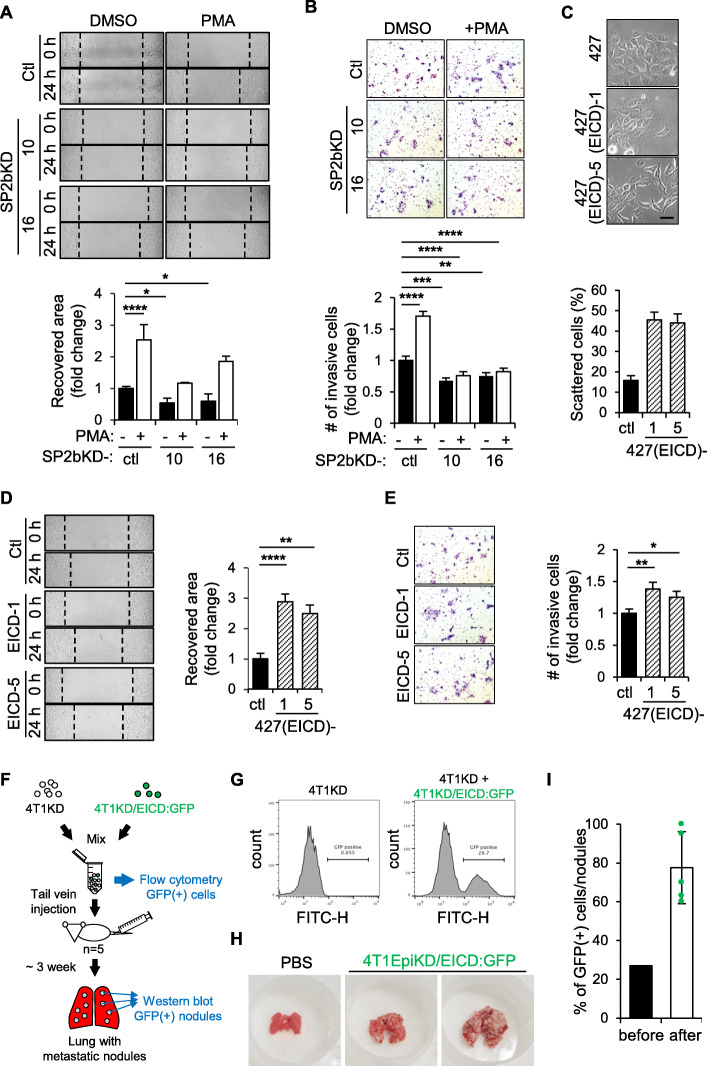
Effects of the intracellular domain of epithin/PRSS14. **a** The wound healing migration of 427, 427(SP2bKD)-10, and -16 cells in PMA-stimulated or non-stimulated conditions was analyzed. Data are presented as the means of the recovered area (six fields per each cell type, *n* = 3). **b** Invasion of 427, 427(SP2bKD)-10 and -16 cells in the presence or absence of PMA was analyzed. Numbers of migrated cells to the other side of the chamber for 24 h were counted, normalized against that of 427 cells, and shown as fold changes (*n* = 3). **c** Representative bright-field images of 427, 427(EICD)-1, and -5 cells are shown. The percentage of scattered cells over the total number of cells (> 745 cells in 50 fields) in each cell type is represented as a bar graph. Scale bars, 50 μm. **d** The degrees of migration of 427, 427(EICD)-1, and -5 for 24 h in the wound healing assay are quantified (six fields per each cell type, *n* = 3) and shown as bar graphs. **e** Invasion of 427, 427(EICD)-1, and -5 cells through transwell pores covered with Matrigel were analyzed as in **b** (5 fields per each cell type, *n* = 3). Statistical analysis was performed using an unpaired two-tailed Student’s *t* test. **f** A diagram of the lung metastasis assay is depicted. **g** The GFP fluorescence of 4T1KD plus EICD-transduced 4T1KD cells (4T1KD/EICD:GFP) was analyzed by flow cytometry and is shown as a histogram. **h** Representative lungs from mice injected with PBS (left) or 4T1KD/EICD:GFP cells (middle, right) are shown. *p < 0.05, **p < 0.01, ***p < 0.001, ****p < 0.0001. Error bars indicate SEM in all panels. **i** The percentage of GFP-positive cells in the mixed cells before injection and the GFP-positive nodules in five lungs after injection are shown. Error bars indicate SD

To establish the existence of EICD, the consequences of its liberation from the membrane were investigated. To this end, 427(SP2bKD) cells were utilized in which shedding-dependent EICD formation is impaired (Fig. 3e). When the ectodomain shedding of epithin/PRSS14 was induced by PMA treatment, 427 cells showed a significant increase in wound healing migration compared to the untreated control (Fig. 4a). PMA treatment also significantly increased the invasion of cells through Matrigel-coated pores of the transwell chamber (Fig. 4b). Knocking down epithin/PRSS14 significantly reduced the PMA-induced migration and invasion (Additional file 1: Figure S6A and S6B). Importantly, the PMA-induced increases in migration and invasion were also largely reduced in 427(SP2bKD) cells (Fig. 4a and b). Moreover, transfection of EICD in the SP2bKD-10 cells rescued the impaired wound healing migration and invasion caused by SPPL2b deficiency (Additional file 1: Figure S6C and S6D). These results suggest that EICD formation from the ectodomain shedding of epithin/PRSS14 may promote cell motility. To directly test the effect of EICD on cell motility, an EICD construct was stably transfected into 427 cells to establish 427(EICD)-1 and -5 cell lines. While the original 427 cells exhibited the epithelial phenotype characterized by well-developed cell-cell contacts forming cohesive sheets of cells, these EICD-expressing cell lines showed loosened cell-cell adhesion and more scattered morphology (Fig. 4c), suggesting that EICD can enhance the motility of the cells. Indeed, migration rates of EICD-expressing cells, compared to the original cells, were significantly increased in the wound healing assay (Fig. 4d) as well as in the Matrigel-coated transwell invasion assay (Fig. 4e). These results prompted the assumption that the well-known enhanced metastatic phenotype of epithin/PRSS14 expressing cancer cells may be caused, at least in part, by the EICD production in the cells. To verify this, epithin/PRSS14 targeting shRNA was stably transfected into 4T1 cells (4T1KD cells), mouse breast cancer cells derived from the BALB/c strain [30], and the cells were transduced with lentivirus encoding EICD followed by the internal ribosome entry site and GFP reporter. The resulting 4T1KD/EICD:GFP cells were injected into BALB/c mice, and the metastatic nodule formation in the lungs was investigated. Comparison of tumor nodule formation between individual mice may cause possible artifacts resulting from the difference in the number of cells injected or in the metastatic susceptibility of individual mice. In order to minimize possible artifacts, these 4T1KD/EICD:GFP cells were mixed with parental control 4T1KD cells in a fixed ratio before injection and the increase in the ratio of EICD-positive tumor nodules to control nodules was analyzed (Diagram, Fig. 4f). In this analysis, EICD-positive cells set as ~ 25% of total cells, verified by flow cytometry before injection (Fig. 4g), made up to about 75% of tumor nodules in the lung (Fig. 4h-i) when the expression of GFP in each nodule was analyzed by Western blot (Additional file 1: Figure S7). Altogether, these results suggest that EICD liberated from the membrane after the ectodomain shedding of epithin/PRSS14 can promote metastatic conversion within the cells.

### EICD induces changes in gene expression

**Fig. 5:**
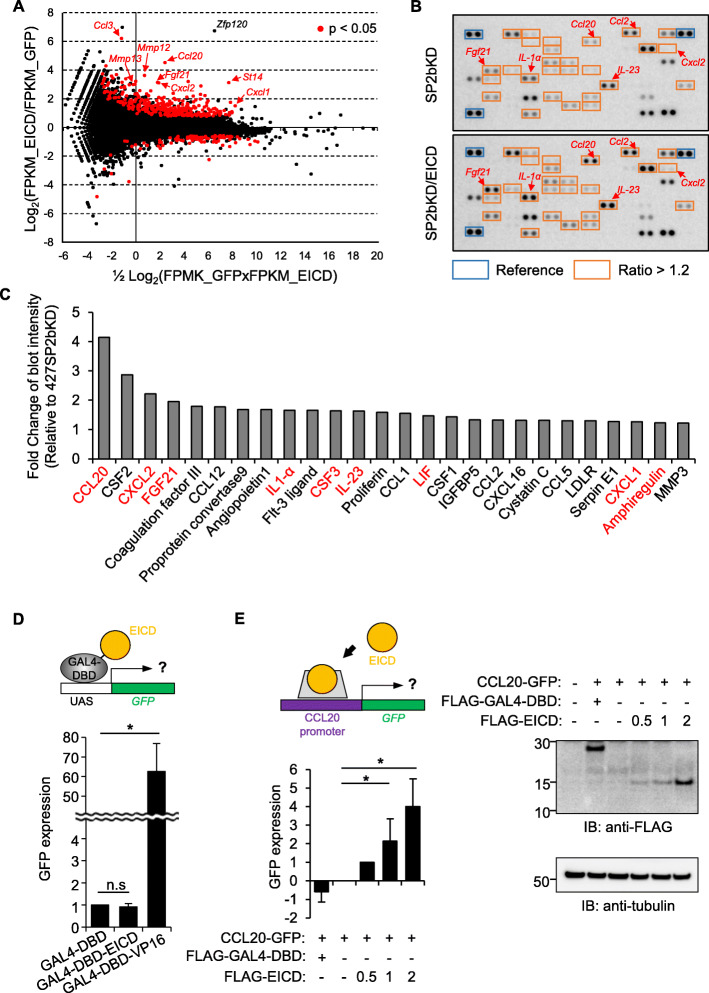
EICD-induced gene expression. **a** DEG analysis was performed between RNA samples from GFP- and GFP plus EICD-transfected cells. The results were visualized as MA plots in which log_2_ fold change (*y*-axis) and mean of normalized counts (*x*-axis) from FPKM (fragments per kilobase million) values are plotted. Genes with *p* values less than 0.05 are shown as red dots. Representative genes are indicated with arrows. **b** Specific antibodies against various cytokines (Additional file 1: Figure S3) were spotted onto the array in duplicate. Conditioned media from SP2bKD-10 cells (SP2bKD, upper) and EICD-expressing SP2bKD-10 cells (SP2bKD/EICD, lower) were incubated on the antibody array. Spots with more than 1.2-fold increases in media from SP2bKD/EICD cells are boxed in red. Positions of representative cytokines were indicated with arrows. **c** Cytokines showing more than a 1.2-fold increase in SP2bKD/EICD cells are shown. Cytokines increased in both the RNA-seq analysis and cytokine array are indicated with red font. **d** The 427 cells were transiently transfected with the GFP gene under the UAS promoter, GAL4 DNA binding domain (GAL4-DBD)-fused EICD, and tdTomato as a transfection marker. The degree of GFP expression in tdTomato-positive cells was analyzed using flow cytometry. The degree of GFP expression in each condition is shown as a bar graph (*n* = 3). Error bars indicate SEM. **e** 427 cells were transfected with GFP gene under CCL20 promoter, EICD, and FLAG-tagged EICD (or FLAG-tagged GAL4-DBD as a control), and tdTomato. The degree of GFP expression of tdTomato positive cells in each condition was subtracted by that of empty vector-transfected cells followed by normalization against that of 0.5 μg FLAG-EICD-transfected cells (left). Expression level of FLAG constructs are shown (right)

Next, the molecular basis of the EICD-mediated increase in cell motility was assessed. Because EICD is found in the nucleus, it was hypothesized that EICD might contain transcription activity as is often observed in other RIP substrates [27]. First, to test this hypothesis, RNA-seq analysis was performed, and the determination of possible genetic changes induced by EICD was attempted. For this purpose, 427(SP2bKD)-10 cells were transfected with either GFP or GFP plus EICD, RNA was prepared from GFP-positive cells, and differentially expressed genes (DEGs) were analyzed between samples. A total of 233 DEGs with more than a twofold increase in EICD-transfected cells were identified (Fig. 5a, red dots) among significant DEGs (*p* value < 0.05, Additional file 2: Table S1). In the gene ontology (GO) analysis using these 233 genes, migration-related GO terms were dominant (Additional file 1: Figure S8), which is consistent with the role of EICD in cell motility. Notably, several chemokine ligands (CCL3, CCL20, and CXCL2), widely studied as mediators of epithelial-mesenchymal transition, tumorigenesis, and metastasis [31–35], were increased (Fig. 5a) and top-ranked in terms of the fold increase (Additional file 2: Table S1). To confirm the effect of EICD on the increase in cytokine mRNA expression, the levels of various cytokines secreted from EICD-expressing SP2bKD cells were checked using a cytokine antibody array (Fig. 5b and Additional file 1: Figure S9). Among the total 111 cytokines tested in the array, the secretion of 26 proteins, such as CCL20 and CXCL2, increased by up to 1.2-fold by EICD expression (Fig. 5c). Among those 26 proteins, the mRNA expression of nine secreted proteins (Fig. 5c, labeled with red fonts) significantly increased in the RNA-seq analysis (Additional file 2: Table S1). This gene expression increase does not appear to result from EICD’s transactivation activity because EICD fused to the Gal4 DNA binding domain did not induce transcription of the UAS-GFP reporter (Fig. 5d) as has been similarly observed in other RIP products, e.g., amyloid precursor protein [36]. Instead, when about 1 kb long promoter region of CCL20, of which expression was increased in both the RNA seq analysis and cytokine array, was inserted upstream of the GFP reporter, it was observed that EICD could significantly induce GFP expression (Fig. 5e), suggesting its possible function in activating machinery involved in the transcription of the CCL20 gene. Altogether, these results showed that EICD liberated from the ectodomain shedding of epithin/PRSS14 can regulate gene expression, especially of cytokines, and modulate surrounding microenvironments favoring cell motility.

### Expression of epithin/PRSS14 and SPPL2B leads to the reduced survival of patients with ER-negative breast cancer

**Fig. 6:**
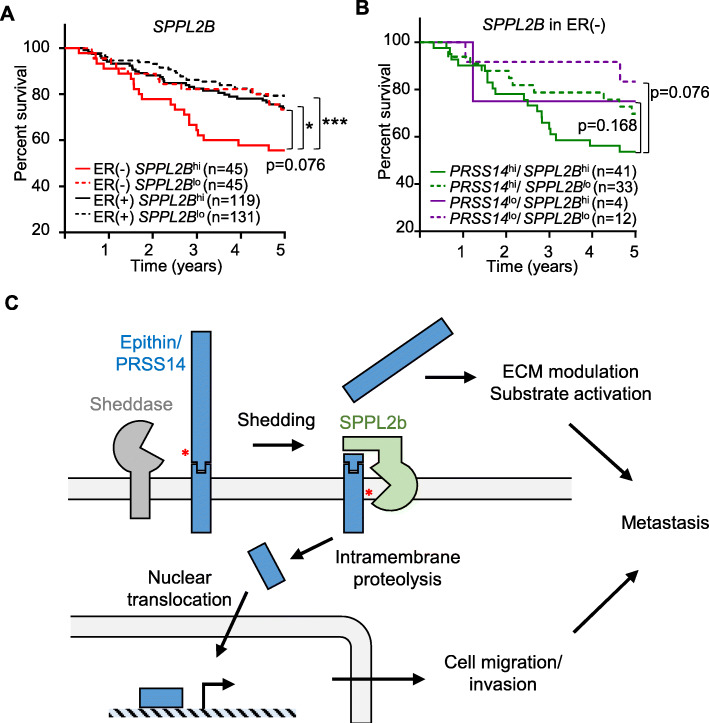
The possible relationship between EICD generation and the prognosis of patients with breast cancer. **a** The Kaplan-Meier survival analysis of four patients with breast cancer grouped by ER status and SPPL2B expression (hi, high; lo, low) is shown. **b** The Kaplan-Meier survival analysis of patients that were ER-negative grouped by expression levels of epithin/PRSS14 and SPPL2B is shown. The *p* values were calculated using log-rank statistics. *p < 0.05; **p < 0.01; ***p < 0.001. **c** A hypothetical model for EICD generation and dual functions of epithin/PRSS14 in metastasis are illustrated

So far, this study indicates that the extracellular protease epithin/PRSS14, upon its shedding and subsequent intramembrane proteolysis, can control intracellular transcriptional events for enhanced cell motility and metastasis. To investigate the clinical relevance of this finding, a bioinformatics approach was utilized. Previously, we reported that high expression of epithin/PRSS14 in patients with ER-negative breast cancer indicated poor patient survival [12]. Consistently, we found that three out of four ER-negative patient samples showed higher expression of epithin/PRSS14 in tumor than in normal tissues (Additional file 1: Figure S10). Interestingly, we also detected the band corresponding to the size of EICD in one patient (Additional file 1: Figure S10, arrow). Considering the additional metastatic potential resulting from EICD formation, it can be assumed that the high expression of SPPL2b may constitute an additional risk factor in patients that are ER-negative. With this hypothesis, the relationship between SPPL2b mRNA levels and the survival of patients with breast cancer was analyzed using The Cancer Genome Atlas data set. In patients that were ER-positive, the expression levels of SPPL2B (high or low, determined based on the median expression level of SPPL2B among those patients) did not show any correlation to the survival of those patients (Fig. 6a). However, in patients with ER-negative breast cancer, the SPPL2B^high^ group showed poorer survival than the SPPL2B^low^ group (Fig. 6a) with the hazard ratio calculated as 1.88 (*p* = 0.076), implying an adverse effect of SPPL2B on the survival of patients with risks from epithin/PRSS14. When the expression levels of both SPPL2B and epithin/PRSS14 were considered in patients that were ER-negative, the epithin/PRSS14^high^SPPL2B^high^ group showed the poorest survival pattern (Fig. 6b), suggesting the clinical significance of the intramembrane proteolysis of epithin/PRSS14. This bioinformatics analysis suggests that the high expression of SPPL2B and epithin/PRSS14 in patients with ER-negative breast cancer may worsen the symptoms, and thus, these can be used as markers for poor prognosis in patients with ER-negative breast cancer.

## Discussion

This study demonstrates that the ectodomain shedding of epithin/PRSS14 induces the SPPL2b-mediated intramembrane proteolysis of NTF, resulting in the release of EICD from the plasma membrane. EICD, in turn, localizes in the nucleus and induces transcriptional changes leading to an increase in gene expression, which possibly enhances cell motility and cancer metastasis (Fig. 6c). Therefore, the shedding of epithin/PRSS14 in cancer cells can result in bidirectional effects; extracellularly, the released protease domain of epithin/PRSS14 can induce the proteolytic modulation of extracellular environments, and intracellularly, the liberated EICD can control gene expression.

The extracellular functions of epithin/PRSS14 have been extensively studied. It has been shown to induce matrix degradation directly through its own proteolytic activity [37] or through the activation of other matrix-degrading enzymes such as urokinase-type plasminogen activators [38] and matrix metalloproteinase-3 [15]. Moreover, epithin/PRSS14 can proteolytically activate growth factors/receptors, including protease-activated receptor 2 [39, 40], hepatocyte growth factor [17], and Tie2 [13], and thus modulate extracellular environments favoring the migration and invasion of cancer or immune cells. Because ectodomain shedding can release most of the extracellular parts of epithin/PRSS14 from the cell surface, it can extend the area affected by these extracellular functions beyond the surface of the cell expressing the protease. Consistent with this, the intracellular functions of epithin/PRSS14, revealed by the current study, are also involved in the modulation of extracellular environments. Intriguingly, most genes, of which expressions are markedly increased by EICD, encode secretory molecules, such as matrix metalloproteinases, cytokines, and growth factors (Fig. 5a), the primary regulators of extracellular environments. In addition, their functions are closely related to cell migration and invasion. For example, CCL20, the expression/secretion of which was most markedly increased by EICD in both the RNA-seq (Fig. 5a) and protein arrays (Fig. 5b), is the ligand of CC chemokine receptor 6 (CCR6) [41] of which signaling has been demonstrated to induce the epithelial-to-mesenchymal transition (EMT), migration, invasion, tumorigenesis, and metastasis of various cancer cells [42–46]. CXCL2, another major cytokine regulated by the presence of EICD (Fig. 5a), is also well-known to promote cancer progression [47] and metastasis [34]. Therefore, it seems that these characteristic bidirectional functions of ectodomain shedding of epithin/PRSS14 can cooperatively facilitate metastasis; its intracellular function can convert primary epithelial tumor cells into migratory mesenchymal carcinoma cells, while its extracellular function allows the degradation of the extracellular matrix and makes a path for the invading cells.

At the moment, however, how EICD can induce transcriptional activation is not currently known. Although the expression of EICD alone sufficiently induced the expression of various genes in the RNA-seq analysis (Fig. 5a) and increased the promoter activity of CCL20 (Fig. 5e), EICD fused to the Gal4-DNA binding domain did not stimulate the promoter activity of the UAS reporter (Fig. 5d), showing that EICD itself does not contain transactivation activity. Therefore, it seems that EICD requires a cofactor(s) to induce specific gene expression and it may control the activity of such cofactors. Promoter analysis using the GeneCards database [48] on genes whose expression increased in both the RNA-seq analysis and cytokine antibody array (Fig. 5c, indicated with red) revealed that several transcription factors, such as IKZF1, POLR2A, CEBPB, MAX, and SP1, are commonly involved in their transcription, suggesting that EICD may cooperate with these transcription factors to induce genetic changes. In this regard, it would be an intriguing future study to investigate the possible cofactors of EICD-mediated intracellular functions. Moreover, the RIP of other type II transmembrane serine proteases [5] and, if present, their differential regulation of gene expression and the mechanism underlying the specific genetic control will be of interest in the future.

## Conclusions

In conclusion, this study reveals that the intracellular domain of epithin/PRSS14 can control gene expression when released from the membrane upon ectodomain shedding of its extracellular domain. This intracellular function cooperates with the extracellular proteolytic functions of the protease can increase cell motility and result in enhanced metastasis. The unexpected function of epithin/PRSS14 revealed by this study strongly suggests that a possible epithin/PRSS14-targeted cancer therapy [6] should consider not only its protease activity but also the intracellular transcriptional function of EICD in tumor progression. More generally, this suggests that similar intramembrane proteolysis possibly occurring in other membrane proteases, such as ADAM10 [49], and their not-yet-identified intracellular functions should be considered when their proteolytic activities are targeted in cancer treatment. Conceptionally, this study also provides a unique example of bidirectional transmembrane signaling in which extracellular functions of a membrane protein are systematically aided by its simultaneous intracellular functions.

## Methods

### Plasmids

To generate FLAG-EICD and FLAG-epithin/PRSS14, epithin/PRSS14 Met1 to Trp55 (EICD) or full length was amplified by PCR and cloned into NotI and BamHI sites of the p3XFLAG-CMV10 vector. For lentiviral infection of EICD, 3XFLAG-EICD was also cloned into a lentiviral vector, pRRLSIN.cPPT.PGK-IRES-GFP.WPRE, modified from pRRLSIN.cPPT.PGK-GFP.WPRE (plasmid ID 12252; Addgene). Human SPPL2b cDNA was purchased from OriGene. UAS-GFP reporter was generated by cloning EGFP cDNA into pHR_5x Gal4 UAS vector (Addgene, #79119). CCL20-GFP reporter constructs were purchased from GeneCopoeia (MPRM34109-LvPF02). cDNA encoding SPPL2b tagged with C-terminal HA was generated by PCR. Epithin/PRSS14 N-terminal end from Met1 to Ala44 fused with GAL4-VP16, EICD-GAL4-VP16, were generated by PCR and inserted into the pM vector (Clontech). The DNA sequence corresponding to Lys45-Val855 of epithin/PRSS14 was inserted into the 3′ of VP16 in the Epi (1-44)-GAL4-VP16 construct, to generate full-length Epi-GAL4-VP16. Epi-GAL4-VP16-strep construct was generated by cloning of PCR-amplified Epi-GAL4-VP16 into pcDNA3.1 vector containing Strep coding sequence. For transient knockdown of epithin/PRSS14, two epithin/PRSS14-specific siRNAs, Epi-1 (target sequence: GCTTGCTGGTGTGGCACTTCCATTA) and Epi-2 (target sequence: GGTGCGCTTCAAACTCTTC), and a universal control siRNA (target sequence: TTCTCCGAACGTCTCACG) were purchased from GenePharma.

### Cell lines and antibodies

427, 427(epiKD), and 4T1KD cells were maintained as described previously [13]. To generate 427(SP2bKD)-10 and -16 cells, shRNA against mouse SPPL2b (target sequence: CCGAGCAGCCTCCAAGTGA) cloned in pSUPER (Oligoengine) was transfected into 427 cells with pcDNA3, selected for antibiotic (G418) resistance, and single-cell clones derived from the antibiotic-resistant pool were tested for their SPPL2b mRNA expression using qPCR. To generate the 427(EICD) cell lines, the intracellular domain of epithin/PRSS14, Met1-Trp55 (EICD) cloned in p3XFLAG-CMV10 vector, p3XFLAG-CMV10/EICD was transfected into 427 cells, selected for antibiotic resistance, and single-cell clones stably expressing EICD were examined by Western blot using the anti-FLAG antibody. Anti-epithin/PRSS14 antibodies, anti-N (against intracellular domain) (WB, 1:500; ICC, 1:100), anti-C (against extracellular domain) (ICC, 1:100), and mAb5 (WB, 1:200), were previously described [18]. Anti-matriptase antibody (IM1014, Milipore, RRID:AB_2255240) (WB, 1:1000), anti-FLAG antibody (clone M2, F1804, Sigma, RRID:AB_262044) (WB, 1:1000; ICC, 1:200), anti-HA antibody (Y-11, sc-805, Santa Cruz Biotechnology, RRID:AB_631618) (WB, 1:1000; ICC, 1:100), anti-Strep antibody (2-1507-001, IBA, RRID:AB_513133), and anti-β-tubulin antibody (T4026, Sigma, RRID:AB_477577) (WB, 1:1000) were purchased. Anti-hN antibody against intracellular domain of human epithin/PRSS14 was raised in rabbits injected with human epithin/PRSS14 peptide (Gly2-His26) and further purified by affinity to recombinant protein, 6xHis-tagged intracellular domain of human epithin/PRSS14.

### Immunocytochemistry

Cells grown on gelatin-coated slide covers were fixed with 3.7% paraformaldehyde in PBS for 10 min, permeabilized with 0.1% TritonX-100 in PBS (PBS-T) for 30 min, and incubated in blocking solution (10% goat serum, 1% gelatin, and 0.1% TritonX-100 in PBS) for 30 min. After washing with PBS-T, samples were treated with a primary antibody in blocking solution for 1 h. Cells then were washed with PBS-T and incubated with FITC-conjugated secondary antibodies (Jackson ImmunoResearch, 711-095-152, 715-095-150, 711-025-152, and 715-025-150) for 50 min. The coverslips were mounted with DAPI (VECTASHIELD, H-1500) and observed under the fluorescence microscope (Axioplan200 M, Carl Zeiss). Images were processed in Photoshop CS6 (Adobe). For quantification of nuclear dots stained by anti-N antibody, a threshold level was set as 1.5 times the mean fluorescence of pixels of which intensities are less than top 60% fluorescence intensity of each nuclear region. If the mean fluorescence of a particular pixel and the 8 pixels surrounding it in the nuclear image is greater than the threshold, the pixel was considered as an EICD-positive pixel. The sum of the fluorescence intensity of EICD-positive pixels in each cell divided by the mean fluorescence of nuclear region of the cell was calculated and represented as normalized EICD nuclear localization. This procedure was automatically performed by a custom Matlab code.

### Cell-based luciferase reporter assay

To examine the liberation of the intracellular domain of epithin/PRSS14, 427 cells were transfected with Epi (1-44)-GAL4-VP16, Epi-GAL4-VP16, or empty vector with GAL4-UAS-Luciferase and pCMV-β-galactosidase (Promega). At 24 h after transfection, these cells were starved for serum and stimulated with PMA/serum and/or ecotin. Cells were lysed, and the luciferase assay was performed according to the manufacturer’s instructions (Promega). Luciferase and β-galactosidase activities were measured using a MicroLumat Plus LB 96 V (Berthold technologies), and the luciferase activity was normalized by β-galactosidase activity.

### Biochemical detection of epithin/PRSS14 fragments

Serum starved 427 cells on a 100-mm dish were treated with 20 μM TAPI-0 (Calbiochem, 579050) for 2 h, DAPT (Sigma, 565770) for 16 h, or (Z-LL)_2_ketone (Calbiochem, 421050) for 16 h. Then, cells were further incubated with 1 μM PMA (Sigma, P1585) for additional 2 h. Whole media were harvested and concentrated using trichloroacetic acid and analyzed for Epi-S′, as described previously [21]. Ice-cold PBS washed cells were lysed with lysis buffer (50 mM HEPES (pH 7.4), 150 mM NaCl, 1% NP-40, 0.5% Sodium deoxycholate, and 1 mM EDTA in the presence of protease inhibitors, leupeptin, aprotinin, and pepstatin. The immunoprecipitation of the PRSS14 N-terminus was performed by antibodies and protein A-Sepharose bead mixtures for 2 h, at 4 °C. The immunoprecipitates were analyzed by SDS-PAGE using 4–12% NuPAGE gradient gel (Invitrogen), followed by Western blot. For the competition assay, 300 ng of N peptide _12_GSQDFGAGLKYNSR_25_ was pre-incubated with anti-N antibody.

### Subcellular fractionation assay

Cells were incubated with serum-free media for 2 h and treated 1 μM PMA for additional 2 h. Those cells were harvested in a lysis buffer for subcellular fractionation (50 mM Tris-HCl, pH 7.5, 10 mM NaCl, 1 mM MgCl_2_, 2 mM EDTA, and protease inhibitors) and passed through a 26-G needle on ice. The nuclear pellets were collected after centrifuge at 700*g* for 10 min at 4 °C. The supernatants were centrifuged at 10,000*g* for 10 min to discard contamination of nuclear fraction and re-centrifuged at 100,000*g* for additional 1 h. The supernatants were collected as cytosolic fraction. The nuclear pellets were resuspended with nuclear lysis buffer (50 mM Tris-HCl, pH 8.0, 150 mM NaCl, 1% NP-40, 0.5% sodium deoxycholate, and protease inhibitors), incubated for 1 h on rotator at 4 °C, and clarified by centrifugation at 17,000*g* for 20 min. Resulting cytosolic and nuclear fractions were analyzed by Western blot.

### Wound healing and invasion assays

For the wound healing assays, cell monolayers maintained in a serum-free medium were scratched using a P200 pipet tip. Cells were washed to remove cell debris and incubated in 1% serum-containing medium for 24 h. Photographs were taken at 0 and 24 h after wounding using Axiovert 200M, and the area covered by migrating cells was determined by the ratio of the scratch area at 0 h to the scratch area at 24 h using ImageJ Software. The invasion assay was performed using a BioCoat Matrigel Invasion Chamber (Corning, 354,480) according to the manufacturer’s instructions. The 3 × 10^5^ cells in the serum-free medium were added into the upper chamber. The lower chamber was filled with DMEM containing 2% serum with or without 1 μM PMA. After 24 h of incubation, the cells on the upper surface of the membrane were removed using cotton swabs. The invaded cells on the lower surface of the membrane were fixed with 100% methanol for 10 min and stained with 0.2% crystal violet for 5 min. The invaded cells were counted under Axioimager M1, and 15 fields were counted. The total number of cells was divided by the number of counted fields in each assay.

### Metastasis assay

4T1KD cells and 4T1KD/EICD:GFP cells were mixed at approximately 7:3 ratio and analyzed by flow cytometry to confirm the ratio. Approximately 5 × 10^5^ cells from the mixture in 100 μl PBS were injected into the tail vein of 5-week-old BALB/c mice (*n* = 5). Three weeks after injection, all mice were sacrificed by cervical dislocation and their lungs were removed and frozen. Each lung tumor nodules from the mice were lysed with RIPA buffer and analyzed for EICD expression using western blot.

### RNA-sequencing and data analysis

FLAG-EICD was co-transfected with pEGFP-c1 (Clontech) into SP2bKD-10 cells. At 24 h after transfection, GFP-positive cells were sorted using FACSAria II (BD Bioscience). The collected GFP-positive cells were immediately lysed with TRIzol (Thermo Fisher Scientific, 15596026), and RNA was extracted. The quality of extracted RNA was verified using an Agilent 2100 BioAnalyzer. RNA-seq was performed at TheragenEtex Bio Institute (Suwon, Korea) using Illumina HiSeq 2500 paired-end RNA sequencing. Reads were aligned with Tophat (v2.1.1) [50], and Fragments per kilobase of exon per million reads (FPKMs) were calculated using Cufflinks (v2.1.1) [51]. Cuffdiff (v2.1.1) [52] was used for normalization. Before generating the MA plot, 0.001 was added to the FPKM values of the EICD-transfected sample and genes with FPKM values less than 0.01 were discarded. For ontology analysis of the EICD-induced gene set, the DAVID functional annotation tool [53, 54] and REVIGO [55] were used.

### Cytokine array

SP2bKD-10 or SP2KD/EICD cells were incubated with 0.5% serum containing DMEM for 24 h. Then, conditioned medium (CM) were concentrated 10 times by centrifugation at 4000 rpm for at 4 °C using ultrafiltration units (Amicon Ultra-15 Centrifugal Filter Unit, 3 kDa cutoff, Millipore). The concentrated CMs were analyzed to determine the expression levels of secreted cytokines by Proteome Profiler Mouse XL Cytokine Array (R&D Systems), according to the manufacturer’s instructions. The signals were detected by ImageQuant LAS 4000 Mini (GE Healthcare), and the pixel density was analyzed by imageJ software.

### Data analyses using TCGA

TCGA data were downloaded using the Broad Institute’s Firehose, a web portal site that has been developed aiming to deliver automated analyses of the TCGA data to general users. For expression correlation analysis, expression values of all patients were drawn in scatter plots with linear interpolation curves between the two genes. The correlation coefficient *r* values between two genes were calculated using the Pearson correlation, and unpaired two-tailed Student’s *t* test was performed. For the 5 years survival rate, Kaplan-Meier survival analysis was used using TCGA breast cancer data excluded data whose contacts were lost in 5 years. The *p* values were calculated using a log-rank (Mantel-Cox) test, and the hazard ratio (HR) was determined by the Mantel-Haenszel method.

## Supplementary information

**Additional file 1: Figure S1.** Surface localization of Epi-GV fusion protein. (A) Diagram of Epi-GV construct with C-terminal Strep tag (Epi-GV-strep). (B) Epi-GV-strep construct (or MOCK) was transfected to 293F cells with a trace amount of EGFP cDNA as a transfection marker, and its surface expression was analyzed by flow cytometry. Representative dot plots are shown. **Figure S2.** PMA-induced nuclear localization of epithin/PRSS14 intracellular domain. (A) 427 cells were transfected with two siRNAs against epithin/PRSS14, starved for serum, and stimulated with PMA or not. Cells were then stained with anti-N antibody (green) and DAPI (blue). Representative images are shown. Scale bars, 20 μm. (B) The computational method for determining EICD-positive pixels is illustrated. If the mean fluorescence of a particular pixel and the 8 pixels surrounding it in the nuclear image is greater than the threshold described in Methods section, the pixel was considered as an EICD-positive pixel. (C) Representative anti-N antibody-stained original image and binary image with epithin/PRSS14 positive pixels indicated in white are shown. (D) In the experiment described in A, normalized EICD nuclear localization (the sum of the fluorescence intensity of EICD-positive pixels in each cell divided by the mean fluorescence of nuclear region of the cell) was calculated and represented as bar graph. At least 57 cells from three microscopic fields were used for each condition. The error bar indicates SEM. ****p < 0.0001 (unpaired two-tailed Student’s *t* test). **Figure S3.** Localization of EICD in the nuclear fraction. (A) 427 cells transfected with specific siRNAs for epithin/PRSS14 and stimulated with PMA as in Fig. 2b. EICD in each condition was detected by immunoprecipitation and subsequent Western blot. CS, control serum. (B) 427 cells were prepared as in (A), the presence of EICD in cytosolic and nuclear fractions was determined by western blot using anti-N antibody. Arrowhead and arrow indicate NTF and EICD, respectively. GAPDH (empty arrowhead) and Histone H3 (asterisk) were used for cytosolic and nuclear marker, respectively. Media and whole cell lysate were also analyzed for epithin/PRSS14 expression using mAb5 with tubulin expression as an internal loading control. **Figure S4.** Effects of γ-secretase inhibitor and SPPL family inhibitor on intramembrane proteolysis of epithin/PRSS14. The 427 cells were treated with indicated concentration of DAPT or (Z-LL)_2_ketone for 16 h, and the spontaneous EICD generation was detected by immunoprecipitation and Western blot using anti-N antibody. Arrowhead and arrow indicate NTF and EICD, respectively. **Figure S5.** Reduced nuclear localization of EICD in SPPL2b-knockdown cell lines. (A) EICD nuclear localization in 427 wild type and SP2bKD-10 cells shown in Fig. 3g was quantified as Figure S2D. At least 73 cells from three microscopic fields were used for each cell type. The error bar indicates SEM. ****p < 0.0001 (unpaired two-tailed Student’s *t* test). (B) The localization of EICD in cytosolic and nuclear fraction of SP2bKD cells was analyzed as in S3B. Arrowhead and arrow indicate NTF and EICD, respectively. GAPDH (empty arrowhead) and Histone H3 (asterisk) were used for cytosolic and nuclear marker, respectively. **Figure S6.** Epithin/PRSS14 and EICD-dependent cell motility. (A) 427 cells were transfected with siRNAs against epithin/PRSS14, and their wound healing migration was tested as in Fig. 4a. Data are presented as the means of the recovered area (six fields per each cell type, *n* = 3). (B) 427 cells were transfected with siRNAs against epithin/PRSS14, and their invasion through Matrigel-coated pores of the transwell chamber was analyzed as in Fig. 4b. Numbers of invaded cells to the other side of the chamber for 24 h were counted, normalized against that of 427 cells, and shown as bar graphs (five fields per each cell type, *n* = 3). (C) Wound healing migration of 427, SP2bKD-10, and EICD-transfected SP2bKD-10 cells was analyzed as in (A). Data are presented as the means of the recovered area (six fields per each cell type, *n* = 3). (D) Transwell invasion of 427, SP2bKD-10, and EICD-transfected SP2bKD-10 cells was analyzed as in (B). Numbers of invaded cells to the other side of the chamber for 24 h were counted, normalized against that of 427 cells, and shown as bar graphs (five fields per each cell type, *n* = 3). Error bars indicate SEM in all panels. *p* < 0.05, **p < 0.01, ***p < 0.001, ****p < 0.0001 (unpaired two-tailed Student’s *t* test). **Figure S7.** Western blot analysis of tumor nodules. (A) Twenty tumor nodules from each lung of five mice injected with 4 T1 and 4T1KD/EICD:GFP mixture were isolated, lysed, and analyzed by Western blot to detect GFP expression. Western blot using anti-tubulin antibodies were used as loading controls in the same blot. GFP-positive nodules are indicated with red font. (B) In case GFP expression was not evident due to the small size of the nodules and the subsequent low protein concentration in the sample, e.g., lanes 2, 15, 17 from mouse #4, the Western blot was repeated using negative (4T1KD) and positive control cells (4T1KD/EICD:GFP). **Figure S8.** Ontology analysis of the EICD-induced gene set. The gene ontology (GO) of total 233 DEGs with more than two-fold increases in EICD-transfected cells was analyzed as in methods. The top ten ranked GO terms were indicated with -log(*p* value). **Figure S9.** Cytokine array. Positions of various cytokine antibodies spotted in the array are shown. **Figure S10.** EICD in human breast cancer patients. The normal (N) and tumor (T) tissues from four ER-negative breast cancer patients were lysed and analyzed by Western blot with anti-human C-terminal (IM1014) and anti-human N antibodies. Arrowhead and arrow indicate NTF and EICD, respectively.

**Additional file 2: Table S1.** Differentially expressed genes (DEG) in the control and EICD-transfected cells. DEGs with *p* values less than 0.05 from the RNA-seq analysis are listed with gene information, FPKMs in the control and EICD-transfected cells, the binary logarithm of the ratio (M), and the geometric mean (A).

## Data Availability

The RNA-seq data generated and analyzed during the current study are available in the NCBI GEO repository (http://www.ncbi.nlm.nih.gov/geo), under the accession number GSE114602. The TCGA datasets used and analyzed during the current study are available from the Broad Institute TCGA Genome Data Analysis Center (2016) web portal site. We used Level 3 normalized RNA-seq data from the Broad GDAC Firehose 2016_01_28 standard data run, 10.7908/C11G0KM9) [56].
