# Supplementary material for: Intramembrane proteolysis of an extracellular serine protease, epithin/PRSS14, enables its intracellular nuclear function
Source: BMC Biol. 2020 Jun 3;18:60. doi: 10.1186/s12915-020-00787-3 (PMC7271384; doi:10.1186/s12915-020-00787-3)
Supplement: Supplementary file 1 — Additional file 1: Figure S1. Surface localization of Epi-GV fusion protein. (A) Diagram of Epi-GV construct with C-terminal Strep tag (Epi-GV-strep). (B) Epi-GV-strep construct (or MOCK) was transfected to 293F cells with a trace amount of EGFP cDNA as a transfection marker, and its surface expression was analyzed by flow cytometry. Representative dot plots are shown. Figure S2. PMA-induced nuclear localization of epithin/PRSS14 intracellular domain. (A) 427 cells were transfected with two siRNAs against epithin/PRSS14, starved for serum, and stimulated with PMA or not. Cells were then stained with anti-N antibody (green) and DAPI (blue). Representative images are shown. Scale bars, 20 μm. (B) The computational method for determining EICD-positive pixels is illustrated. If the mean fluorescence of a particular pixel and the 8 pixels surrounding it in the nuclear image is greater than the threshold described in Methods section, the pixel was considered as an EICD-positive pixel. (C) Representative anti-N antibody-stained original image and binary image with epithin/PRSS14 positive pixels indicated in white are shown. (D) In the experiment described in A, normalized EICD nuclear localization (the sum of the fluorescence intensity of EICD-positive pixels in each cell divided by the mean fluorescence of nuclear region of the cell) was calculated and represented as bar graph. At least 57 cells from three microscopic fields were used for each condition. The error bar indicates SEM. ****p < 0.0001 (unpaired two-tailed Student’s t test). Figure S3. Localization of EICD in the nuclear fraction. (A) 427 cells transfected with specific siRNAs for epithin/PRSS14 and stimulated with PMA as in Fig. 2b. EICD in each condition was detected by immunoprecipitation and subsequent Western blot. CS, control serum. (B) 427 cells were prepared as in (A), the presence of EICD in cytosolic and nuclear fractions was determined by western blot using anti-N antibody. Arrowhead and arrow indic [file 12915_2020_787_MOESM1_ESM.pdf]

Figure S1.

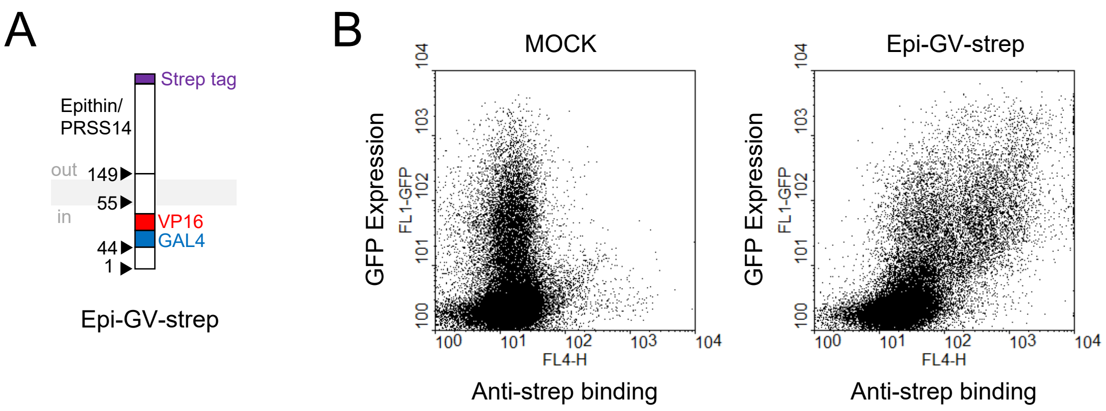
A
Strep tag
Epithin/
PRSS14
out
149
55
in
VP16
GAL4
44
1
Epi-GV-strep
B
MOCK
Epi-GV-strep
GFP Expression
FL1-GFP
FL4-H
Anti-strep binding

Figure S2.

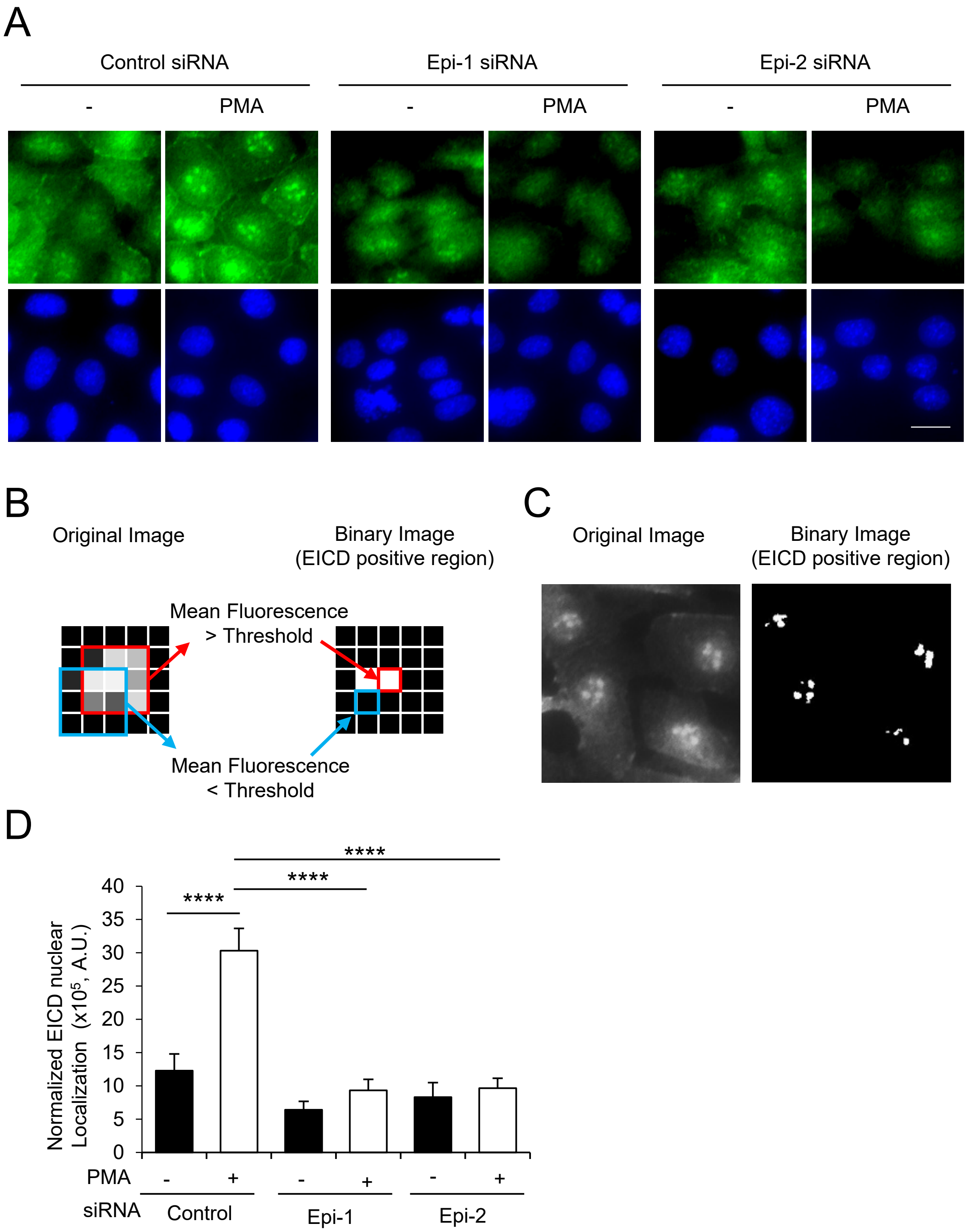
A
Control siRNA
Epi-1 siRNA
Epi-2 siRNA
-
PMA
B
Original Image
Binary Image
(EICD positive region)
Mean Fluorescence
> Threshold
Mean Fluorescence
< Threshold
C
Original Image
Binary Image
(EICD positive region)
D
Normalized EICD nuclear Localization (x10^5, A.U.)
0
5
10
15
20
25
30
35
40
****
****
****
PMA
-
+
siRNA
Control
Epi-1
Epi-2

Figure S3.

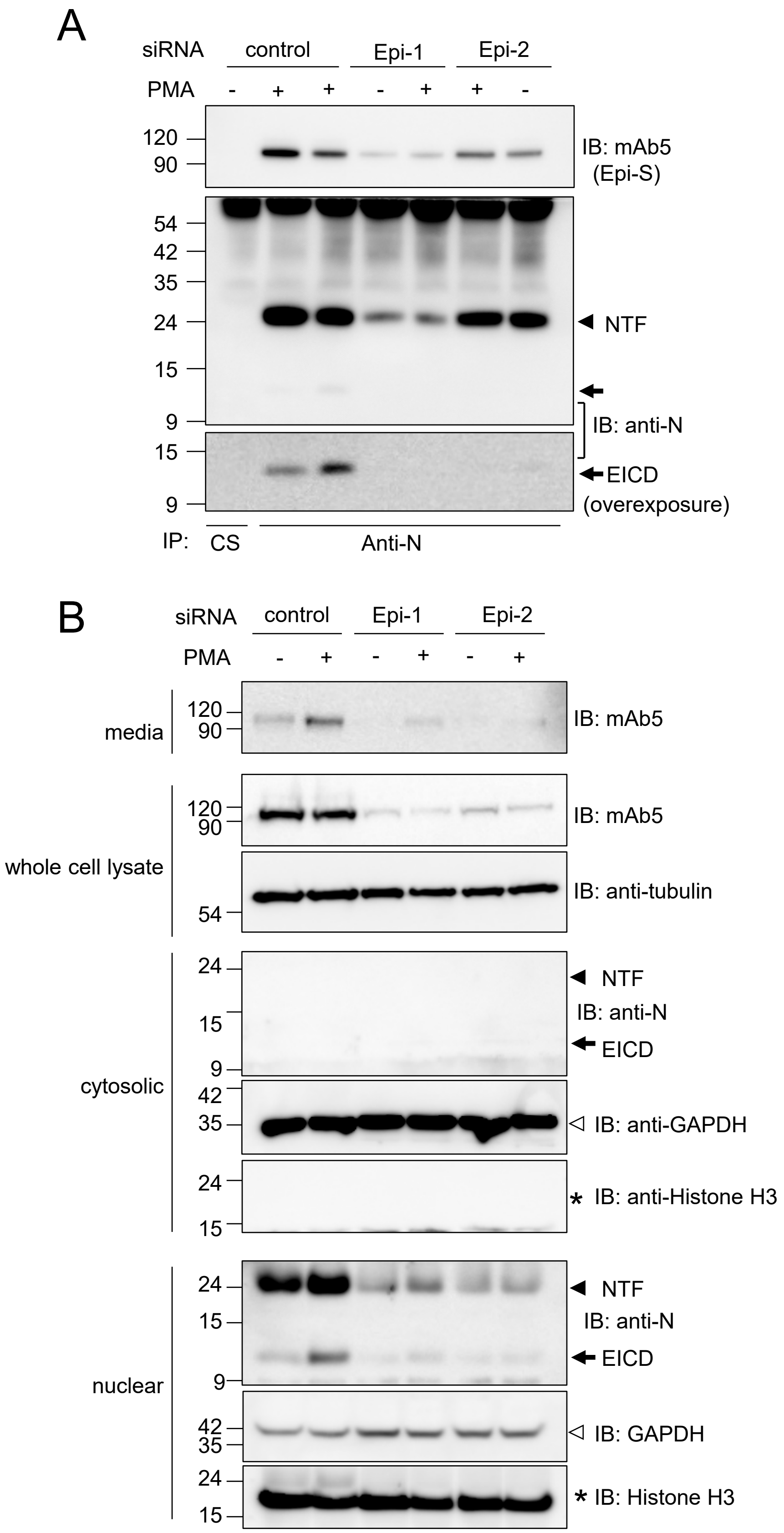
A
siRNA
control
Epi-1
Epi-2
PMA
- + + - + + -
120
90
IB: mAb5
(Epi-S)
54
42
35
24
15
9
NTF
IB: anti-N
15
9
EICD
(overexposure)
IP:
CS
Anti-N
B
siRNA
control
Epi-1
Epi-2
PMA
- + - + - +
media
120
90
IB: mAb5
whole cell lysate
120
90
IB: mAb5
54
IB: anti-tubulin
cytosolic
24
15
9
NTF
IB: anti-N
EICD
42
35
IB: anti-GAPDH
24
15
* IB: anti-Histone H3
nuclear
24
15
9
NTF
IB: anti-N
EICD
42
35
IB: GAPDH
24
15
* IB: Histone H3

Figure S4.

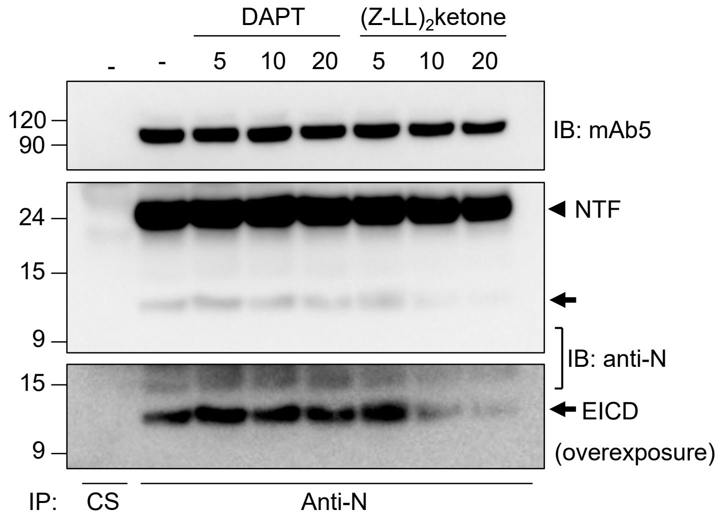

DAPT
(Z-LL)$_2$ketone
- - 5 10 20 5 10 20
120
90
IB: mAb5
24
15
9
NTF
15
9
EICD
IB: anti-N
(overexposure)
IP: CS Anti-N

Figure S5.

A

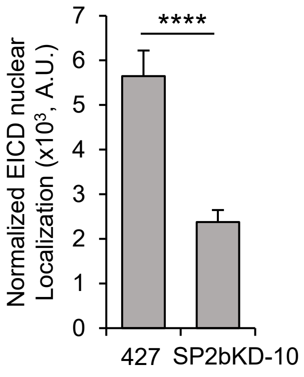
Normalized EICD nuclear Localization (x10³, A.U.)
****
0
1
2
3
4
5
6
7
427
SP2bKD-10

B

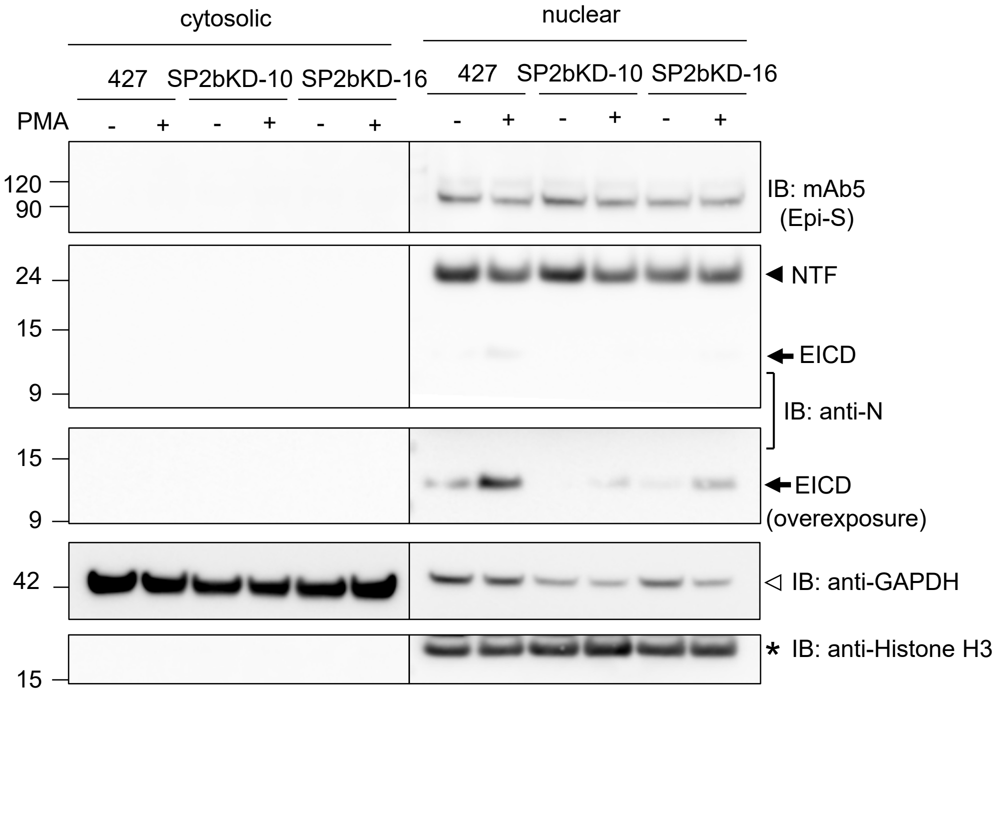
cytosolic
nuclear
427
SP2bKD-10
SP2bKD-16
PMA
120
90
24
15
9
42
IB: mAb5
(Epi-S)
NTF
EICD
IB: anti-N
EICD
(overexposure)
IB: anti-GAPDH
IB: anti-Histone H3

Figure S6.

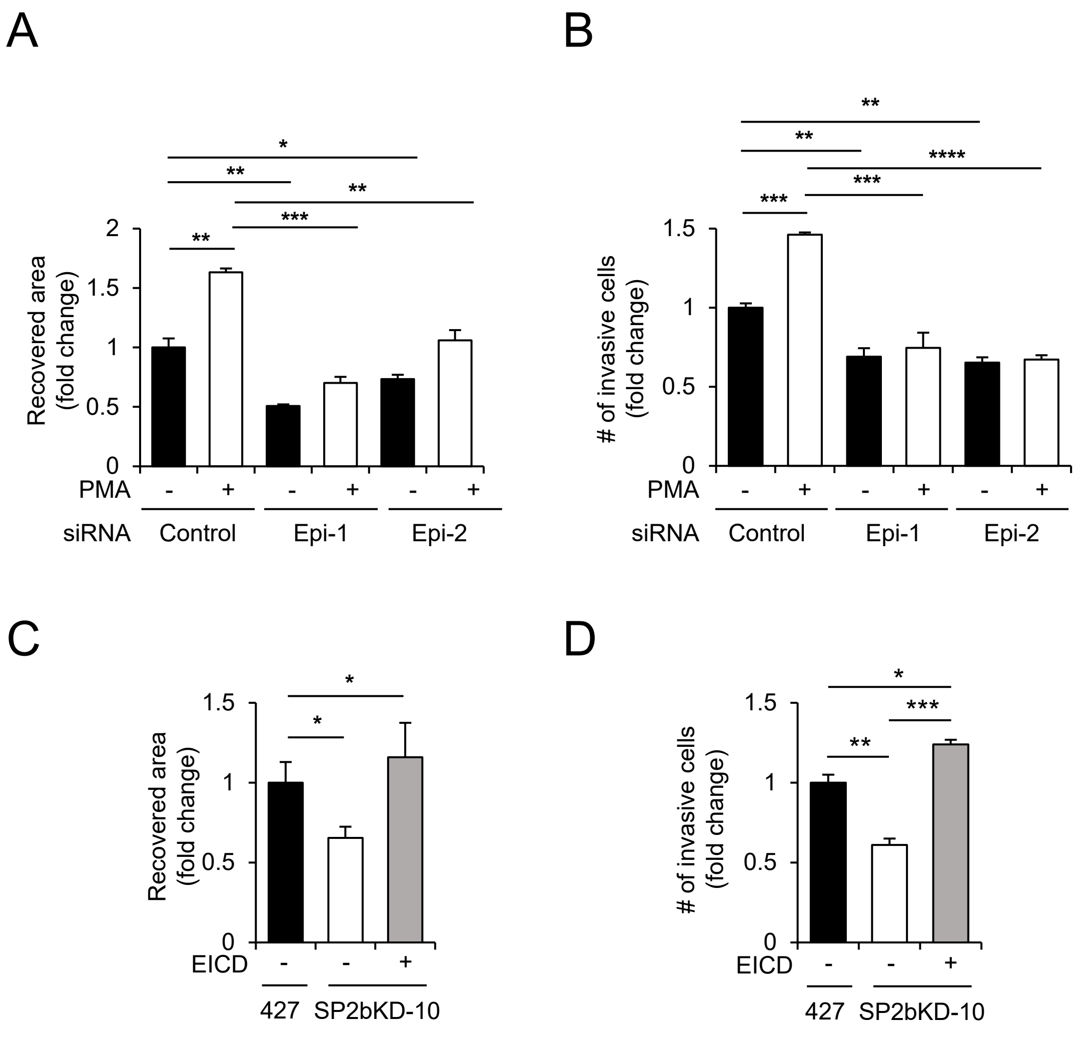
A
Recovered area (fold change)
2
1.5
1
0.5
0
*
**
**
***
**
PMA
-
+
-
+
-
+
siRNA
Control
Epi-1
Epi-2
B
# of invasive cells (fold change)
1.5
1
0.5
0
**
**
****
***
***
PMA
-
+
-
+
-
+
siRNA
Control
Epi-1
Epi-2
C
Recovered area (fold change)
1.5
1
0.5
0
*
*
EICD
-
-
+
427
SP2bKD-10
D
# of invasive cells (fold change)
1.5
1
0.5
0
*
***
**
EICD
-
-
+
427
SP2bKD-10

Figure S7.

A

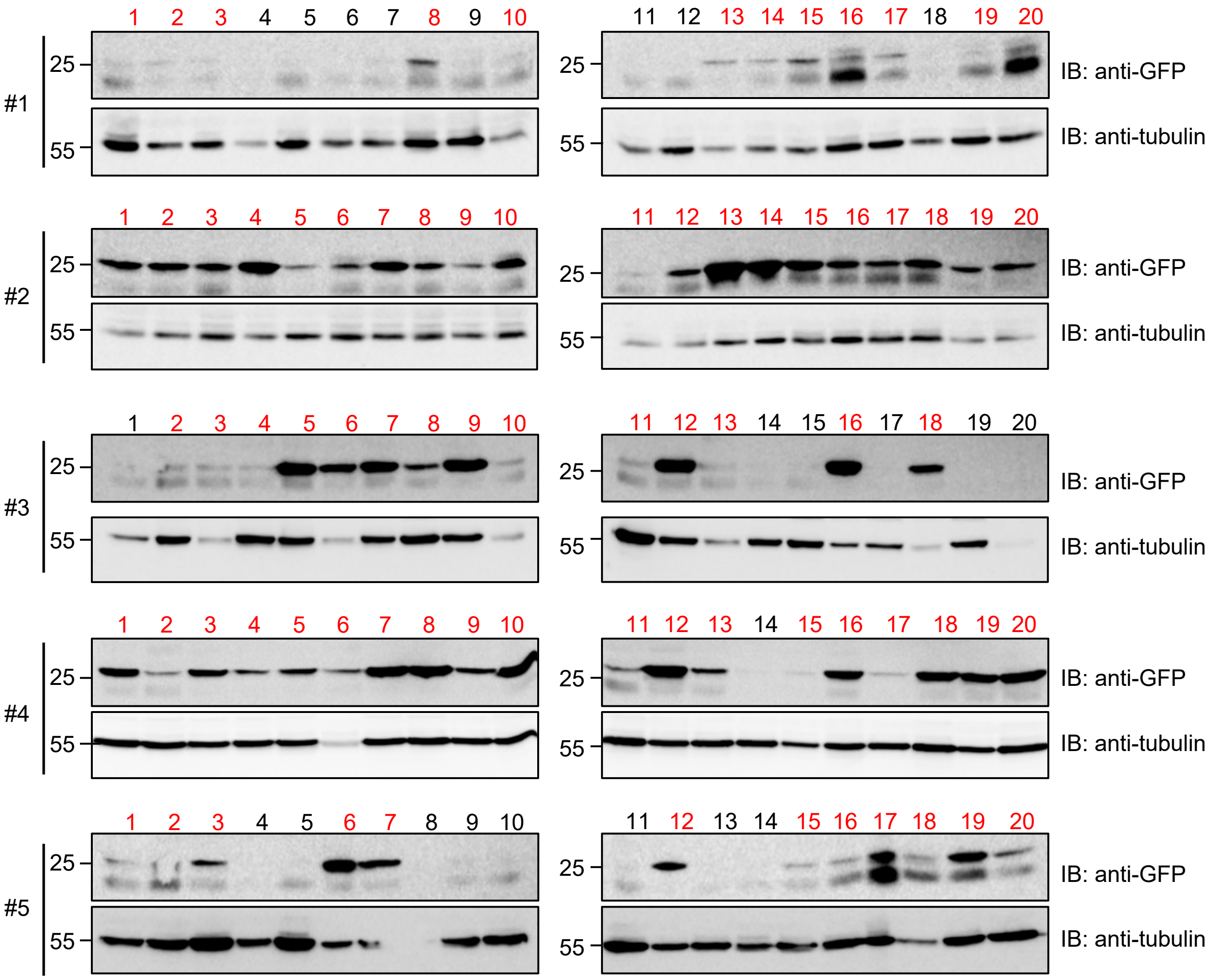
1 2 3 4 5 6 7 8 9 10
11 12 13 14 15 16 17 18 19 20
#1
#2
#3
#4
#5
25
55
IB: anti-GFP
IB: anti-tubulin

B

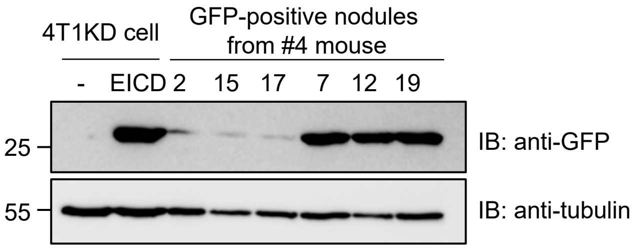
4T1KD cell
GFP-positive nodules from #4 mouse
- EICD 2 15 17 7 12 19
25
55
IB: anti-GFP
IB: anti-tubulin

Figure S8.

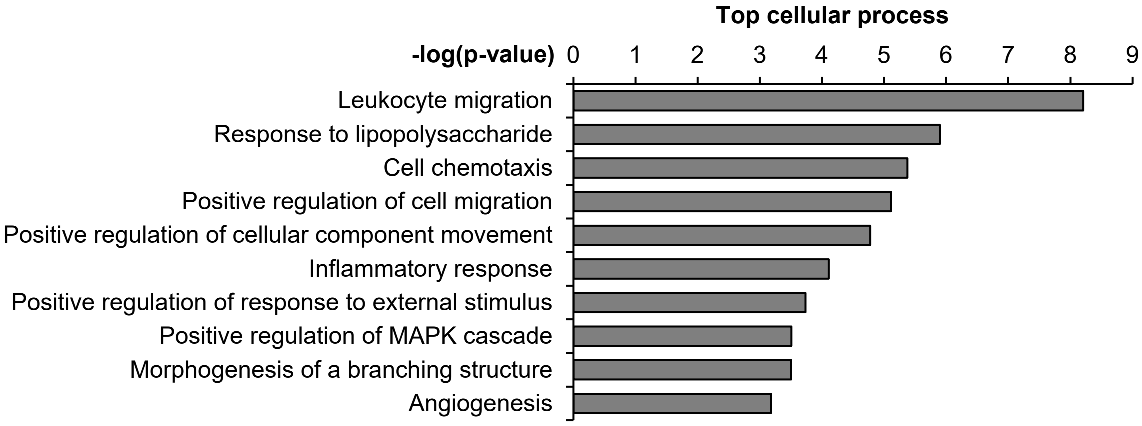
Top cellular process
-log(p-value)
0
1
2
3
4
5
6
7
8
9
Leukocyte migration
Response to lipopolysaccharide
Cell chemotaxis
Positive regulation of cell migration
Positive regulation of cellular component movement
Inflammatory response
Positive regulation of response to external stimulus
Positive regulation of MAPK cascade
Morphogenesis of a branching structure
Angiogenesis

Figure S9.

	1 2	3 4	5 6	7 8	9 10	11 12	13 14	15 16	17 18	19 20	21 22	23 24
A	Reference	Acrp30	Amphiregulin	Angiopoietin1	Angiopoietin2	Angiopoietin-like3	TNFSF13B	C1qR1	CCL2	CCL3	CCL5	Reference
B		CCL6	CCL11	CCL12	CCL17	CCL19	CCL20	CCL21	CCL22	CD14	TNFRSF5	
C		CD160	Chemerin	Chitinase 3-like1	Coagulation Factor III	C5a	Compement Factor D	C-Reactive Protein	CX3CL1	CXCL1	CXCL2	
D	CXCL9	CXCL10	CXCL11	CXCL13	CXCL16	Cystatin C	DKK1	DPPIV	EGF	Endoglin	Endostatin	Fetuin A
E	FGF acidic	FGF21	Flt-3 Ligand	Gas6	CSF3	GDF15	CSF2	HGF	ICAM1	IFN-γ	IGFBP1	IGFBP2
F	IGFBP3	IGFBP5	IGFBP6	IL-1α	IL-1β	IL-1ra	IL-2	IL-3	IL-4	IL-5	IL-6	IL-7
G	IL-10	IL-11	IL-12 p40	IL-13	IL-15	IL-17A	IL-22	IL-23	IL-27 p28	IL-28A/B	IL-33	LDLR
H	Leptin	LIF	Lipocalin2	LIX	CSF1	MMP2	MMP3	MMP9	Myeloperoxidase	Osteopontin	TNFRSR11B	PD-ECGF
I	PDGF-BB	Pentraxin2	Pentraxin3	Periostin	Pref-1	Proliferin	Proprotein Convertase9	RAGE	RBP4	Reg3G	Resistin	
J	Reference	E-Selectin	P-Selectin	Serpin E1	Serpin F1	Thrombopoietin	TIM-1	TNFα	VCAM-1	VEGF-A	WISP1	Neg Control

Figure S10.

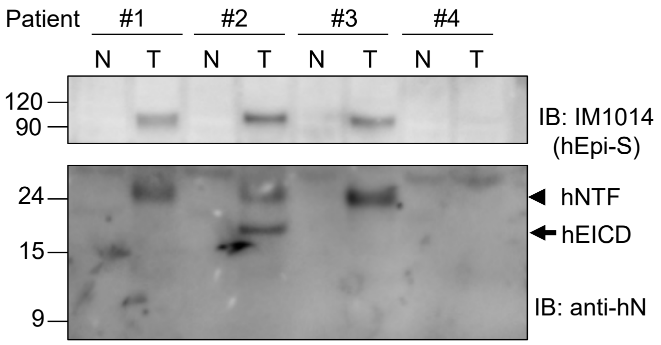
Patient
#1
#2
#3
#4
N
T
N
T
N
T
N
T
120
90
IB: IM1014
(hEpi-S)
24
15
9
hNTF
hEICD
IB: anti-hN
